# CT-based radiomics nomogram of vertebral bodies and paravertebral muscles for differential diagnosis of type 2 diabetes mellitus

**DOI:** 10.3389/fendo.2026.1763664

**Published:** 2026-03-27

**Authors:** Xuewei Zheng, Xiaolong Qu, Minghui Sun, Jun Ding, Boyao Sun

**Affiliations:** 1 Department of Radiology, China-Japan Union Hospital of Jilin University, Changchun, China; 2 Department of Radiology, Tianjin Cancer Hospital Airport Hospital, Tianjin, China; 3 Department of Radiology, Chaoyang District People’s Hospital of Changchun City, Changchun, China; 4 Department of Gastrointestinal and Colorectal Surgery, China-Japan Union Hospital of Jilin University, Changchun, China

**Keywords:** computed tomography, paravertebral muscles, radiomics, type 2 diabetes mellitus, vertebral body

## Abstract

**Background:**

Diabetes mellitus has emerged as a global public health concern, boasting a high prevalence rate worldwide. Given this situation, accurately identifying type 2 diabetes mellitus (T2DM) holds great significance as it plays a pivotal role in enabling early intervention and facilitating more effective management of the disease. Against this backdrop, it becomes essential to explore the diagnostic value of radiomics features extracted from CT images of vertebral bodies (VB) and paravertebral muscles (PVM) in relation to type 2 diabetes mellitus (T2DM).

**Methods:**

A total of 160 cases of clinical and imaging data were retrospectively collected, including 80 patients with T2DM and 80 non-diabetic patients. Regions of interest (ROIs) of VB and PVM were delineated for all subjects, and radiomics features were extracted. Patients were divided into a training group (n=112) and a validation group (n=48) at a 7:3 ratio. Key radiomics features of VB and PVM were screened using independent samples t-test and least absolute shrinkage and selection operator (LASSO) algorithm. A k-nearest neighbor (KNN) classifier was used to establish radiomics models based on VB and PVM, and radiomics scores (Rad-scores) were calculated by weighting the coefficients of the selected features. Clinical risk factors were identified via univariate and multivariate logistic regression to construct a clinical model. A nomogram was then developed by integrating the Rad-score with the clinical model using multivariate logistic regression. The diagnostic performance of the models was evaluated using the area under the receiver operating characteristic curve (AUC), calibration curves, and clinical decision curves, with the Delong’s test applied to compare performance among models.

**Results:**

In the training set, the AUCs of the VB radiomics model, PVM radiomics model, VB-PVM combined radiomics model, clinical model, and radiomics-clinical combined model were 0.902, 0.948, 0.952, 0.857, and 0.956, respectively; in the validation set, the corresponding AUCs were 0.873, 0.880, 0.894, 0.758, and 0.926. The radiomics-clinical combined model showed the best diagnostic performance. Calibration and decision curves indicated that the radiomics nomogram had good consistency and clinical applicability.

**Conclusion:**

The combined radiomics and clinical model based on CT images of VB and PVM has good diagnostic value for the differential diagnosis of T2DM.

## Introduction

T2DM is an endocrine and metabolic disorder involving disturbances in carbohydrate, protein, and fat metabolism, caused by absolute or relative insulin deficiency and reduced insulin sensitivity in target cells (1). During diabetes management, we have observed changes in body composition among many patients, including alterations in bone, muscle, fat, and vascular calcification (2). Osteoporosis and sarcopenia, as emerging complications of diabetes, have attracted increasing attention in recent years (3). Changes in skeletal muscle health can affect systemic glucose homeostasis, as skeletal muscle, together with the liver, plays a key role in regulating glucose uptake and maintaining glucose balance (4–6).

In patients with T2DM, both hyperglycemia and insulin resistance can lead to reductions in muscle tissue, strength, and mass, accompanied by increased intramuscular fat infiltration (7). Compared with non-diabetic individuals, T2DM patients exhibit decreased paravertebral muscle mass and vertebral bone density as the disease progresses. Meanwhile, the proportion of undiagnosed diabetes worldwide remains high (8–10). Thus, there is a need to develop new approaches for early diagnosis and differential identification of the disease. Pathological changes in the VB and PVM of T2DM patients can be evaluated using radiological techniques such as CT and MRI. Radiomics, an emerging field in medical imaging, uses advanced computational methods to extract high-dimensional data from standard imaging modalities, providing valuable insights for tumor characterization (11, 12). Radiomics approaches can overcome the limitations of visual image assessment by quantifying features of regions of interest, and these quantifications play a crucial role in diagnosis, clinical prognosis, treatment selection, and decision support (13–15).

In this study, we evaluated the diagnostic efficacy of CT radiomics features of VB and PVM in distinguishing T2DM patients from non-diabetic individuals, aiming to provide a novel method for T2DM diagnosis.

## Materials and methods

This study was conducted in accordance with the Declaration of Helsinki and approved by the ethics committee of China-Japan Union Hospital of Jilin University(approval No. 2024122607). All patients signed an informed consent form for inclusion in the study. It included 80 patients with confirmed diabetes mellitus and 80 non-diabetic patients who underwent plain computed tomography (CT) scanning of the entire abdomen between January 2024 and January 2025.

### Patients

**Figure 1:**
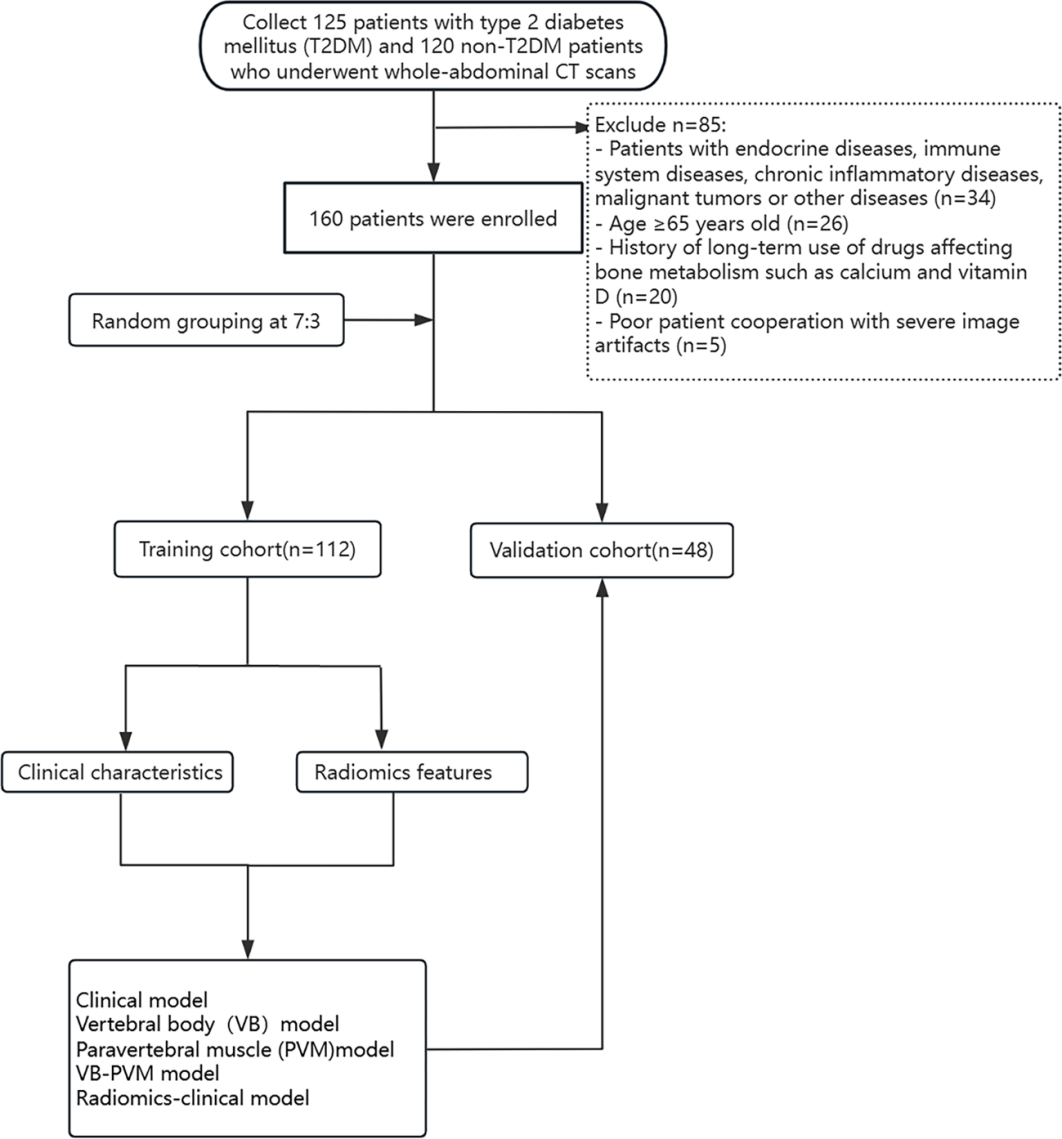
Flowchart of study patients.

We retrospectively collected 125 patients with type 2 diabetes mellitus (T2DM) and 120 non-T2DM patients who attended China-Japan Union Hospital of Jilin University from January 2024 to January 2025, and recorded their basic data. After screening based on the inclusion and exclusion criteria, a total of 160 patients were finally enrolled in the study, with their age, gender, and body mass index (BMI) recorded (**Figure 1**).

Inclusion criteria for T2DM patients:(1) They should meet the 1999 WHO diagnostic criteria for diabetes (16). (2) Aged between 20 and 65 years to exclude the impact of excessively old or young age on bones and muscles.(3) Having undergone whole abdominal CT scanning during the visit.

Exclusion criteria for T2DM patients:(1) Other types of diabetes: such as type 1 diabetes, gestational diabetes, etc.(2) Patients with other diseases such as endocrine diseases, immune system diseases, chronic inflammatory diseases, and malignant tumors;(3) A history of long-term use of drugs affecting bone metabolism such as calcium and vitamin D.

Inclusion criteria for non-T2DM patients:(1) No confirmed history of diabetes, and no receipt of any antihyperglycemic drugs (e.g., metformin, insulin, etc.) or insulin sensitizers within the past year.(2) Normal glucose indicators: fasting blood glucose < 6.1 mmol/L, 2-hour oral glucose tolerance test (OGTT) < 7.8 mmol/L, glycated hemoglobin (HbA1c) < 5.7%, with all indicators tested within the past 3 months.(3) Age and gender: age-matched with the diabetes group, and the gender ratio as close as possible to reduce the impact of age and gender factors on vertebral bodies and paravertebral muscles.

Exclusion criteria for non-T2DM patients: (1) Complicated with major diseases: such as malignant tumors, severe infections, liver and kidney failure, active stage of autoimmune diseases, etc. (2) A history of long-term use of drugs affecting bone metabolism such as calcium and vitamin D.

All enrolled T2DM patients were diagnosed with the disease for more than 2 years and received regular metformin-based hypoglycemic therapy with standardized regimens. Specifically, 17 cases were administered monotherapy, 41 cases received dual-drug combination therapy, 11 cases underwent triple or more oral hypoglycemic drug combination therapy, and 11 cases received insulin therapy (basal insulin or premixed insulin alone, or in combination with oral hypoglycemic agents).

### Clinical data collection

The age, gender, and BMI of each patient were recorded. The clinical data included the average CT values of the 12th thoracic vertebra (T12), the 1st lumbar vertebra (L1), and the bilateral psoas major muscles (PM). Due to its relatively large area and ease of measurement, the CT value of the psoas major muscle was selected as a clinical factor in this study. When measuring the CT values of T12 and L1, the cortical bone and posterior venous plexus should be avoided. The size of the region of interest (ROI) was approximately 100 mm^2^ when measuring the CT values of the vertebrae and psoas major muscles.

### Radiomics feature extraction

**Figure 2:**
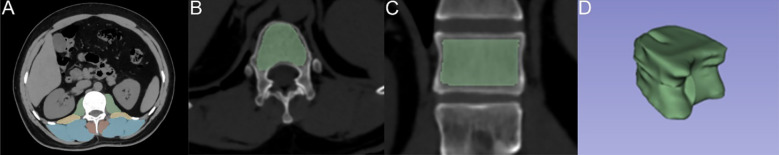
The delineation process of ROIs for paravertebral muscles and vertebral bodies. In **(A)**, the psoas major is indicated in green, the quadratus lumborum in yellow, the multifidus in red, and the erector spinae in blue. **(B–D)** demonstrate the delineation of the ROI in the 12th thoracic vertebra (T12).

All CT images of T2DM patients and non-T2DM patients were imported into the software in DICOM format. We used 3D Slicer software (version 5.4.0) to manually draw the ROIs of VB and PVM on CT images for segmentation. Two radiologists with 5 years of experience in CT diagnosis manually outlined the contours of the psoas major muscle (PM), quadratus lumborum muscle (QL), erector spinae muscle (ES), and multifidus muscle (MF) on CT images (**Figure 2**). Under the background of bone window, the ROI was placed within the cancellous bone of the T12 vertebra, and manual outlining was performed along the inner edge of the vertebral cortical bone. During this process, the outlining range was strictly controlled to ensure that adjacent intervertebral discs and pedicles were not included (**Figure 2**). When there was wedge deformation or other lesions in the T12 vertebra of patients, the L1 vertebra was outlined instead. To evaluate the inter-observer and intra-observer repeatability of ROI contour outlining, 50 patients’ images were randomly selected after an interval of 2 weeks, and one of the doctors repeated the ROI segmentation to assess the intra-observer repeatability. A good consistency was considered when the intraclass correlation coefficient (ICC) > 0.75.

For both the original images and wavelet-transformed images, a total of 851 radiomics features were extracted for each patient. Specifically, these features included 162 first-order features, 14 morphological features, and 675 texture features. Among them, the texture features were further subdivided into multiple categories, including 216 gray-level co-occurrence matrix (GLCM) features, 126 gray-level dependence matrix (GLDM) features, 144 gray-level run-length matrix (GLRLM) features, 144 gray-level size zone matrix (GLSZM) features, and 45 neighboring gray-tone difference matrix (NGTDM) features.

### Feature selections and radiomics score construction

Prior to the following analyses, all extracted features of each patient were standardized using z-score. Firstly, the stability of the features was evaluated using the ICC. Features with an ICC > 0.75 were considered to have strong repeatability and reliability and were included in the subsequent study. Secondly, independent sample t-tests were used to select the most relevant radiomics features from the vertebrae and paravertebral muscles for differential diagnosis, while excluding irrelevant and redundant features. Finally, the least absolute shrinkage and selection operator (LASSO) algorithm was used to identify key radiomics features. Subsequently, the optimal parameters were determined through 10-fold cross-validation. The key radiomics features selected from the VB and PVM were used to construct a Rad-score, which was calculated for each patient by linearly combining the selected features weighted by their respective coefficients.

### Model construction and assessment

Univariate logistic regression analysis and multivariate logistic regression analysis were performed on clinical variables to identify independent predictors significantly associated with T2DM, thereby constructing a clinical model. As shown in **Tables 1**, **2**, among the included clinical variables (age, gender, BMI, vertebral body CT value, paravertebral muscle CT value), only vertebral body CT value and paravertebral muscle CT value were identified as independent risk factors for T2DM with statistical significance (all P < 0.05) in both univariate and multivariate logistic regression analyses, while age, gender and BMI showed no significant correlation with T2DM (all P > 0.05) and were thus excluded from the final clinical For each predictor, the odds ratio (OR) was calculated as a measure of relative risk, along with the 95% confidence interval (CI). Next, prediction models were established using the optimal radiomics features screened from the VB and PVM, as well as independent clinical risk factors. These models included the VB model, PVM model, VB-PVM muscle model, clinical model, and radiomics-combined clinical model. The combined models were visualized as nomograms via multivariate logistic regression. The predictive accuracy of the five models was evaluated using the area under the AUC. The Delong test was conducted to compare differences in the ROCs of the five models. Calibration curves were used to assess the consistency between predicted risks and observed outcomes. Decision curve analysis (DCA) was performed to evaluate the net benefit and clinical utility of the models.

**Table 1:** The clinical characteristics in the training cohort and validation cohort.

Variables	Training cohort (n=112)			Validation cohort (n=48)			P
	T2DM	Non-T2DM	P	T2DM	Non-T2DM	P	
Gender			0.329			0.148	0.075
Male	n=38	n=32		n=14	n=8		
Female	n=18	n=24		n=10	n=16		
Age	55.32 ± 12.01	51.13 ± 11.75	0.114	52.21 ± 8.63	48.93 ± 13.14	0.101	0.623
BMI (kg/m^2^)	25.00 ± 4.19	24.5 ± 2.67	0.453	24.13 ± 3.12	23.87 ± 2.95	0.768	0.526
Vertebral body CT value	113.36 ± 38.72	164.06 ± 58.95	<0.001	118.94 ± 31.44	163.71 ± 56.58	0.006	0.804
Paravertebral muscle CT value	43.53 ± 3.52	47.31 ± 4.04	<0.001	40.04 ± 6.03	47.74 ± 3.92	<0.001	0.084

**Table 2:** Univariate and multivariate analyses of clinical variables in the training cohort.

	Univariate logistic regression		Multivariate logistic regression	
Variables	OR (95%CI)	P	OR (95%CI)	P
Age (years)	1.04(1.00, 1.08)	0.062		
Male, (n%)	1.47(0.62, 3.46)	0.383		
BMI (kg/m^2^)	1.01(0.984,1.042)	0.392		
Vertebral body CT value	0.98(0.97, 0.99)	<0.010*	0.97(0.95, 0.99)	0.010*
Paravertebral muscle CT value	0.73(0.63, 0.85)	<0.010*	0.79(0.65, 0.95)	0.010*

*p < 0.05, statistically significant results from logistic regression analysis.

### Statistical analysis

Statistical analyses were performed using Python (version 3.13.5), with the ‘scikit-learn’ package (version 1.6.1) employed for radiomics and clinical model construction. Independent samples t-tests were conducted for continuous variables, and chi-square tests were used for categorical variables. Quantitative data were expressed as mean ± standard deviation for normally distributed data. Logistic regression analyses and nomogram construction were performed using the ‘rms’ package (version 8.0.0) in R software (version 4.5.1). A two-tailed P-value < 0.05 was considered statistically significant.

## Results

### Clinical characteristics

A total of 160 patients were included in this study, comprising 80 T2DM patients and 80 non-T2DM patients. The gender, age, BMI,VB CT values, and PVM CT values of all patients were recorded. **Table 1** presents the clinical characteristics of the two groups. The patients were divided into a training set (n=112) and a validation set (n=48).

### Feature selection and development of the radiomics signature

**Figure 3:**
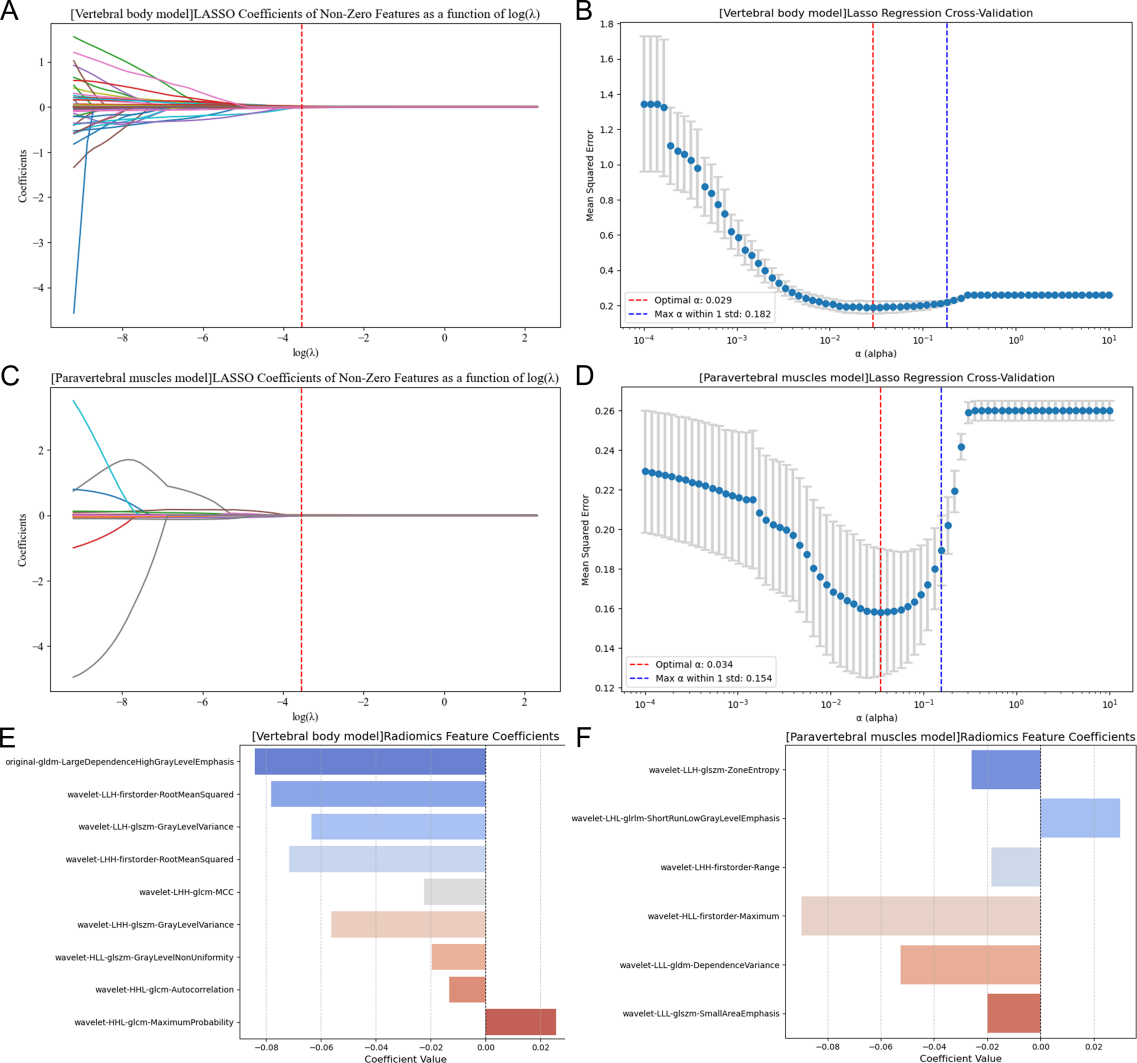
**(A, C)** respectively represent the changes in LASSO coefficients of vertebral body and paravertebral muscle features with the regularization parameter (λ value). **(B, D)** demonstrate the selection of the tuning parameter (λ) in the LASSO models. In this study, the optimal λ values corresponding to the vertical lines were selected, which yielded 9 and 6 features with non-zero coefficients from the radiomic features of vertebral bodies and paravertebral muscles, respectively. **(E, F)** show the contribution of these 9 and 6 features with non-zero coefficients to the radiomic features.

A total of 851 radiomics features were extracted from the CT images of the vertebrae and paravertebral muscles, respectively. First, features with an ICC < 0.75 were excluded. Next, through independent sample t-tests, 127 and 88 features were selected from the radiomics features of the vertebrae and paravertebral muscles, respectively. Then, the parameter λ was adjusted and tested through 10-fold cross-validation of LASSO regression. Finally, 9 key radiomics features were selected from the VB radiomics features, and 6 key radiomics features were selected from the PVM radiomics features (**Figure 3**). The corresponding coefficients were calculated, and the Radscore was computed by weighting the selected features according to their coefficients.

The calculation results of Radscores for VB and PVM are as follows:

VB-Radscore=-0.084 ∗ original-gldm-LargeDependenceHighGrayLevelEmphasis.
-0.078 ∗ wavelet-LLH-firstorder-RootMeanSquared.
-0.064 ∗ wavelet-LLH-glszm-GrayLevelVariance.
-0.072 ∗ wavelet-LHH-firstorder-RootMeanSquared.
-0.022 ∗wavelet-LHH-glcm-MCC.
-0.056∗ wavelet-LHH-glszm-GrayLevelVariance.
-0.020∗ wavelet-HLL-glszm-GrayLevelNonUniformity.
-0.013 ∗ wavelet-HHL-glcm-Autocorrelation.
+ 0.026 ∗ wavelet-HHL-glcm-MaximumProbability.
+ 0.029.
PVM-Radscore = −0.026 ∗ wavelet-LLH-glszm-ZoneEntropy.
+ 0.029 ∗ wavelet-LHL-glrlm-ShortRunLowGrayLevelEmphasis.
-0.018∗ wavelet-LHH-firstorder-Range.
-0.089 ∗ wavelet-HLL-firstorder-Maximum.
-0.053 ∗ wavelet-LLL-gldm-DependenceVariance.
-0.019 ∗ wavelet-LLL-glszm-SmallAreaEmphasis.
+ 0.034.

### Radiomics nomogram construction and the discrimination performance of the different models

**Figure 4:**
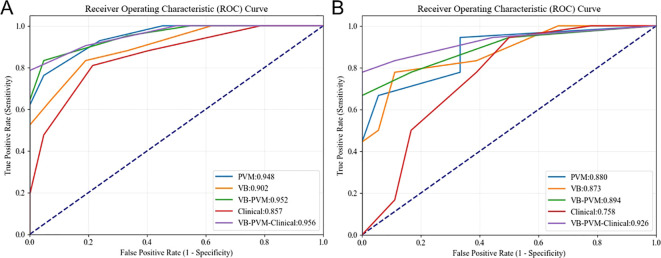
Receiver operating characteristic curves of the five models in the **(A)** training and **(B)** test sets, respectively.

**Figure 5:**
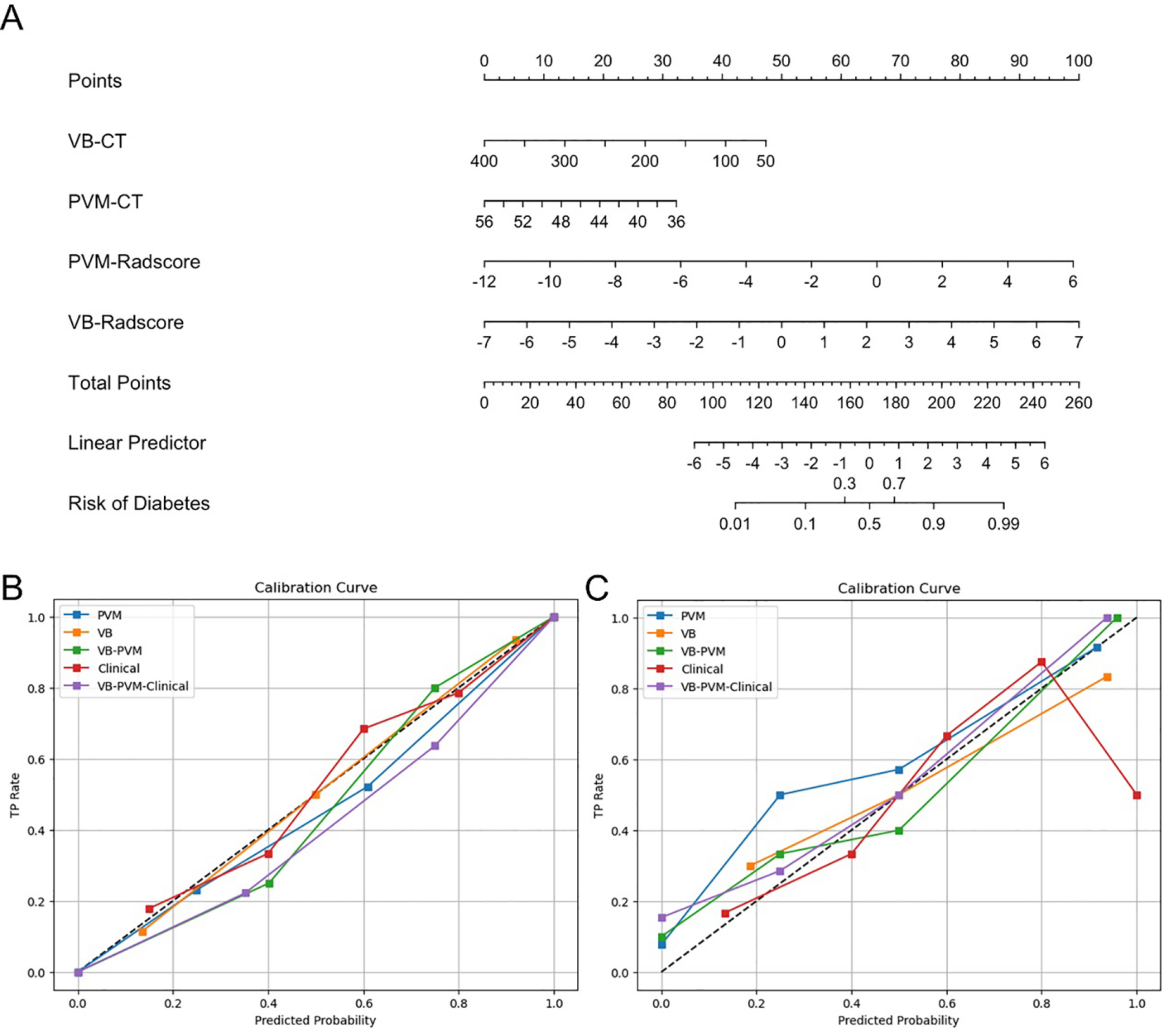
Radiomics nomogram and calibration curves of the five models. **(A)** Radiomics nomogram developed in the training set, which combines vertebral CT value, paravertebral muscle CT value, and radiomics score. **(B, C)** Calibration curves of the five models in the training set **(B)** and test set **(C)**. The dashed line indicates the optimal prediction, and the solid line represents the actual predictive ability of the model. The closer the solid line is to the dashed line, the better the performance of the model.

**Figure 6:**
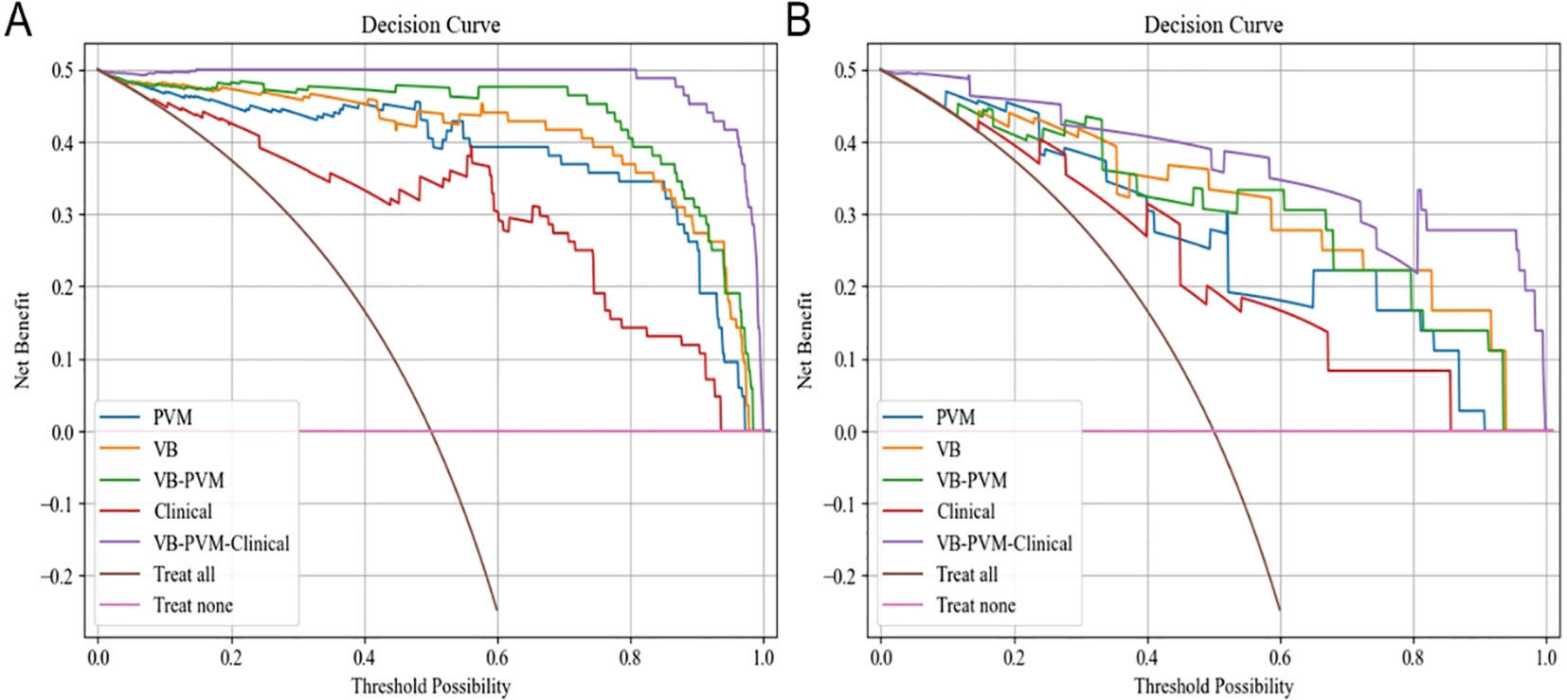
Decision curve analysis of five models. **(A)** Decision curve of the training set. **(B)** Decision curve of the validation set. The y-axis represents net benefit; the x-axis represents threshold probability. The radiomics nomogram demonstrated a higher overall net benefit in distinguishing type 2 diabetes mellitus from non-type 2 diabetes mellitus compared to the clinical factor model and default strategies (i.e., “treat all as type 2 diabetes mellitus” or “treat all as non-type 2 diabetes mellitus”).

**Table 2** presents the results of univariate and multivariate logistic regression analyses. Both univariate and multivariate analyses identified several clinical factors significantly associated with T2DM, including VB CT values and PVM CT values. **Table 3** lists the sensitivity, specificity, accuracy, and AUC of the VB model, PVM model, VB-PVM model, clinical model, and radiomics-combined clinical model. **Figure 4** shows the ROC curves of the five models in the training and testing sets. A nomogram was developed using multivariate logistic regression based on clinical characteristics and Radscores (**Figure 5**). This model incorporates VB CT values, PVM CT values, and radiomics Radscores of VB and PVM. The calibration curves in the training and validation cohorts (**Figure 5**) demonstrated good consistency between the nomogram predictions and the actual patient outcomes. For the testing set, both the radiomics-combined clinical model and the radiomics model exhibited good performance in the differential diagnosis of type 2 diabetes mellitus. The Delong test indicated that in the testing set, the AUC of the radiomics nomogram was significantly higher than that of the clinical model (p = 0.028), while no significant differences were observed in AUC between the remaining radiomics models and the radiomics nomogram (P > 0.05). DCA showed that the radiomics nomogram had a higher overall net benefit than the clinical model across most reasonable threshold probability ranges for identifying patients with type 2 diabetes mellitus (**Figure 6**).

**Table 3:** Diagnostic efficacy of each model and analysis of area under the ROC curve.

Model		AUC (95%CI)	P	Sensitivity	Specificity	Accuracy
VB Model	Training dataset	0.902 (0.833, 0.970)	<0.001	0.833	0.810	0.821
	Test dataset	0.873 (0.754, 0.993)	0.001	0.778	0.889	0.833
PVM Model	Training dataset	0.948 (0.899, 0.998)	<0.001	0.762	0.952	0.857
	Test dataset	0.880 (0.763, 0.996)	0.001	0.667	0.944	0.806
VB-PVM Model	Training dataset	0.952(0.904,1.00)	<0.001	0.833	0.952	0.893
	Test dataset	0.894(0.784,1.00)	0.002	0.667	0.999	0.833
Clinical Model	Training dataset	0.857(0.774,0.938)	<0.001	0.809	0.809	0.809
	Test dataset	0.758(0.599,0.917)	<0.001	0.778	0.611	0.694
VB-PVM-Clinical Model	Training dataset	0.956(0.906,1.00)	<0.001	0.881	0.905	0.893
	Test dataset	0.926(0.834,1.00)	<0.001	0.778	0.997	0.889

## Discussion

In this study, a nomogram integrating CT radiomic features of VB and PVM with clinical factors was constructed, which was confirmed to have excellent diagnostic value for the differential diagnosis of T2DM. The results showed that the CT radiomic features of VB and PVM were both closely associated with T2DM. Moreover, the combined model integrating these features with clinical indicators (including CT values of VB and PVM) achieved the highest diagnostic efficacy, providing a novel non-invasive tool for the early differential diagnosis and screening of T2DM.

Hyperglycemia impairs osteoblast function and promotes osteoclast activity, while insulin resistance may reduce bone formation by affecting the insulin-like growth factor signaling pathway (17, 18). The quantitative alterations in radiomic texture features of the vertebral body in patients with T2DM essentially represent direct imaging manifestations of skeletal microstructural remodeling and disrupted bone–muscle metabolic coupling induced by chronic hyperglycemia and insulin resistance. The core biological basis includes non-enzymatic glycation of vertebral collagen and paravertebral myosteatosis. Under chronic hyperglycemia, collagen, the main organic component of the bone matrix (19), undergoes non-enzymatic glycation, resulting in the formation and excessive accumulation of advanced glycation end products (AGEs) (20, 21). This process disrupts the normal cross-linking architecture of collagen molecules and compromises the mechanical integrity of the bone matrix. Meanwhile, AGEs bind to the receptor for advanced glycation end products (RAGE), thereby inhibiting osteoblastic differentiation and promoting osteoclastic proliferation and activation via upregulation of proinflammatory cytokines such as tumor necrosis factor-α (TNF-α) and interleukin-6 (IL-6) (22). These cascades ultimately lead to decreased vertebral trabecular number, increased trabecular separation, and heterogeneous distribution of cancellous bone density—microstructural alterations that can be accurately quantified by radiomic texture parameters such as the gray level dependence matrix (GLDM) (22, 35). In addition, insulin resistance further suppresses osteoblastic mineralization by downregulating the insulin-like growth factor-1 (IGF-1) signaling pathway, while simultaneously promoting paravertebral myosteatosis (18). Intramuscular fat infiltration and reduced muscle mass lead to insufficient mechanical loading on the vertebral body, which in turn aggravates skeletal microstructural deterioration and forms a pathological bone–muscle metabolic cycle. These changes ultimately present as characteristic alterations in the CT-based radiomic texture features of the vertebral body (23, 24).

Numerous studies have explored the potential of radiomics in predicting issues related to T2DM. Specifically, Wei et al. (25) confirmed that bone radiomics holds significant promise in predicting vertebral fractures in elderly T2DM patients. Similarly, Qiu et al. (1) reported that radiomics based on paraspinal muscle CT imaging exhibits favorable predictive value in detecting abnormal bone mass in T2DM patients. There are also some studies that have employed imaging and machine learning to predict bone health (26–29). However, to our knowledge, no previous studies have reported the combination of VB and PVM features for the differential diagnosis of T2DM. This study is the first to integrate radiomic features of skeletal muscles, including those of VB and PVM, for the differential diagnosis of T2DM. Traditional T2DM screening methods, such as oral glucose tolerance test and glycated hemoglobin detection, have limitations in large-scale population screening due to invasiveness, cost, or compliance issues. Radiomic analysis utilizes image-derived features and enhances the accuracy of diagnosis and treatment by leveraging advanced image analysis technologies as well as the rapid generation and validation of imaging data. It provides a powerful tool for modern medicine (30, 31).

In this study, 9 key features were selected from VB CT images and 6 from PVM images via LASSO regression to construct radiomics models (VB-Radscore and PVM-Radscore). Among these features,*original-gldm-LargeDependenceHighGrayLevelEmphasis* from VB and *wavelet-HLL-firstorder-Maximum* from PVM were identified as the optimal predictors for T2DM. The GLDM-LargeDependenceHighGrayLevelEmphasis feature quantifies the concentration of high gray-level regions over large spatial scales, reflecting the structural heterogeneity within tissues (32). The wavelet-HLL-firstorder-Maximum feature, by extracting vertical high-frequency edge information via wavelet transform, captures the most prominent gray-level changes in images (33). Overall, these comprehensively and meticulously extracted features capture image information from multiple dimensions, providing a rich data foundation for subsequent analyses and model construction.

Given the high heterogeneity of T2DM, subclassification based on core pathophysiological features (insulin resistance, β-cell function, islet autoantibodies) and clinical factors (age at diagnosis, BMI) has become a cornerstone of precision medicine for T2DM (34), and this stratification implies distinct radiomic phenotypes of vertebral body (VB) and paravertebral muscle (PVM) across different subtypes: insulin resistance-dominant subtypes may show more severe PVM fat infiltration with characteristic texture radiomic features, while β-cell dysfunction-dominant subtypes tend to present significant VB bone loss with abnormal trabecular radiomic signatures (35, 36). Our findings of decreased VB/PVM CT values and specific abnormal radiomic features in T2DM patients can be interpreted as the comprehensive reflection of heterogeneous bone and muscle impairments induced by different T2DM pathophysiological subtypes in the cohort, and the high diagnostic efficacy of the VB-PVM combined radiomic model further confirms that integrating bone and muscle radiomic features can capture such subtype-based body composition heterogeneity (36, 37). Regrettably, due to the retrospective design of this study, critical laboratory indicators for formal T2DM subclassification (fasting C-peptide, insulin levels for HOMA-IR, islet autoantibodies) were not systematically collected, which limits subtype-specific radiomic analysis; thus, conducting prospective studies with complete T2DM subtype stratification to explore subtype-specific VB/PVM radiomic features and construct subtype-integrated radiomic nomograms will be our key future research direction (37).

This study has several limitations. First, this was a single-center retrospective study with a relatively small sample size (160 cases), which may introduce selection bias and limit the external validity and generalizability of our findings. Thus, large-scale, multi-center prospective studies are warranted to verify the stability and reproducibility of our model. Second, our cohort was derived from a single medical center and lacked diverse ethnic representation, which may restrict the generalizability of the results to other ethnic populations. Further studies involving multi-ethnic cohorts are needed to strengthen the applicability of our findings.

## Data Availability

The original contributions presented in the study are included in the article/supplementary material. Further inquiries can be directed to the corresponding authors.

## References

[1] Miao S Yu F Sheng R Zhang X Li Y Qi Y . Radiomics of pericoronary adipose tissue on computed tomography angiography predicts coronary heart disease in patients with type 2 diabetes mellitus. BMC Cardiovasc Disord. (2024) 24:300. doi: 10.1186/s12872-024-03970-4, PMID: 38867152 PMC11167783

[2] Dagdeviren S Jung DY Friedline RH Noh HL Kim JH Patel PR . IL-10 prevents aging-associated inflammation and insulin resistance in skeletal muscle. FASEB J. (2017) 31:701–10. doi: 10.1096/fj.201600832R, PMID: 27811060 PMC5240661

[3] Shou J Chen PJ Xiao WH . Mechanism of increased risk of insulin resistance in aging skeletal muscle. Diabetol Metab Syndr. (2020) 12:14. doi: 10.1186/s13098-020-0523-x, PMID: 32082422 PMC7014712

[4] Stump CS Henriksen EJ Wei Y Sowers JR . The metabolic syndrome: role of skeletal muscle metabolism. Ann Med. (2006) 38:389–402. doi: 10.1080/07853890600888413, PMID: 17008303

[5] D’Souza DM Al-Sajee D Hawke TJ . Diabetic myopathy: impact of diabetes mellitus on skeletal muscle progenitor cells. Front Physiol. (2013) 4:379. doi: 10.3389/fphys.2013.00379, PMID: 24391596 PMC3868943

[6] Chadt A Al-Hasani H . Glucose transporters in adipose tissue, liver, and skeletal muscle in metabolic health and disease. Pflugers Arch. (2020) 472:1273–98. doi: 10.1007/s00424-020-02417-x, PMID: 32591906 PMC7462924

[7] Huang S Xiang C Song Y . Identification of the shared gene signatures and pathways between sarcopenia and type 2 diabetes mellitus. PLoS One. (2022) 17:e0265221. doi: 10.1371/journal.pone.0265221, PMID: 35271662 PMC8912249

[8] Marshall SM . The pancreas in health and in diabetes. Diabetologia. (2020) 63:1962–5. doi: 10.1007/s00125-020-05235-z, PMID: 32894305

[9] Abbasi A Sahlqvist AS Lotta L Brosnan JM Vollenweider P Giabbanelli P . A systematic review of biomarkers and risk of incident type 2 diabetes: an overview of epidemiological, prediction and aetiological research literature. PLoS One. (2016) 11:e0163721. doi: 10.1371/journal.pone.0163721, PMID: 27788146 PMC5082867

[10] Fisher-Hoch SP Vatcheva KP Rahbar MH McCormick JB . Undiagnosed diabetes and pre-diabetes in health disparities. PLoS One. (2015) 10:e0133135. doi: 10.1371/journal.pone.0133135, PMID: 26186342 PMC4505949

[11] Mayerhoefer ME Materka A Langs G Häggström I Szczypiński P Gibbs P . Introduction to radiomics. J Nucl Med. (2020) 61:488–95. doi: 10.2967/jnumed.118.222893, PMID: 32060219 PMC9374044

[12] Lim HK Ha HI Park SY Han J . Prediction of femoral osteoporosis using machine-learning analysis with radiomics features and abdomen-pelvic CT: A retrospective single center preliminary study. PLoS One. (2021) 16:e0247330. doi: 10.1371/journal.pone.0247330, PMID: 33661911 PMC7932154

[13] Zhou Y Zhang J Chen J Yang C Gong C Li C . Prediction using T2-weighted magnetic resonance imaging-based radiomics of residual uterine myoma regrowth after high-intensity focused ultrasound ablation. Ultrasound Obstet Gynecol. (2022) 60:681–92. doi: 10.1002/uog.26053, PMID: 36054291 PMC9828488

[14] An C Kim DW Park YN Chung YE Rhee H Kim MJ . Single hepatocellular carcinoma: preoperative MR imaging to predict early recurrence after curative resection. Radiology. (2015) 276:433–43. doi: 10.1148/radiol.15142394, PMID: 25751229

[15] Tallam H Elton DC Lee S Wakim P Pickhardt PJ Summers RM . Fully automated abdominal CT biomarkers for type 2 diabetes using deep learning. Radiology. (2022) 304:85–95. doi: 10.1148/radiol.211914, PMID: 35380492 PMC9270681

[16] Grimaldi A Heurtier A . Critères diagnostiques du diabète de type 2 [Diagnostic criteria for type 2 diabetes]. Rev Prat. (1999) 49:16–21. 9926711

[17] Koromani F Oei L Shevroja E Trajanoska K Schoufour J Muka T . Vertebral fractures in individuals with type 2 diabetes: more than skeletal complications alone. Diabetes Care. (2020) 43:137–44. doi: 10.2337/dc19-0925, PMID: 31658976 PMC7411280

[18] Lin HH Hsu HY Tsai MC Hsu LY Chien KL Yeh TL . Association between type 2 diabetes and osteoporosis risk: A representative cohort study in Taiwan. PLoS One. (2021) 16:e0254451. doi: 10.1371/journal.pone.0254451, PMID: 34255803 PMC8277062

[19] Shi P Gong H Lyu L Liu S Jia S Li C . Low bone turnover is associated with advanced glycation end-products, oxidative stress, and inflammation induced by type 2 diabetes mellitus. FASEB J. (2024) 38(15), e23871. doi: 10.1096/fj.202400790R, PMID: 39109498

[20] Daroux M Prévost G Maillard-Lefebvre H Gaxatte C D'Agati VD Schmidt AM . Advanced glycation end-products: implications for diabetic and non-diabetic nephropathies. Diabetes Metab. (2010) 36, 1–10. doi: 10.1016/j.diabet.2009.06.005, PMID: 19932633

[21] Zhou M Archibeck ES Feteih Y Abubakr Y O'Connell GD . Non-enzymatic glycation increases the failure risk of annulus fibrosus by predisposing the extrafibrillar matrix to greater stresses. Acta Biomater. (2023) 168, 223–234. doi: 10.1016/j.actbio.2023.07.003, PMID: 37433360

[22] Muschitz C Kautzky-Willer A Winhofer Y Rauner M Haschka J Cejka D . Diagnose und Management der Osteoporose bei Diabetes mellitus (Update 2023): Gemeinsame Leitlinie der Österreichischen Gesellschaft für Knochen- und Mineralstoffwechsel und der Österreichischen Diabetes Gesellschaft. Wien Klin Wochenschr. (2023) 135(Suppl 1), 207–224. doi: 10.1007/s00508-022-02118-8, PMID: 37101043 PMC10133052

[23] Visser M Goodpaster BH Kritchevsky SB Newman AB Nevitt M Rubin SM . Muscle mass, muscle strength, and muscle fat infiltration as predictors of incident mobility limitations in well-functioning older persons. J Gerontol A Biol Sci Med Sci. (2005) 60, 324–333. doi: 10.1093/gerona/60.3.324, PMID: 15860469

[24] Oliveira LB Silva VO Jucá ICL Torres JVGST Torres MRS Moura F . Myosteatosis and Type 2 Diabetes Mellitus. Acta Med (Hradec Kralove). (2025) 68(2), 37–44. doi: 10.14712/18059694.2025.17, PMID: 41064888

[25] Wei Z Yu R Zhang Y Wang Y Wang J Xie C . A CT-based radiomic model for predicting vertebral fractures in older patients with type 2 diabetes mellitus: A longitudinal study. J Endocrinol Invest. (2025) 48, 2123–2135. doi: 10.1007/s40618-025-02627-z, PMID: 40493166

[26] Chu S Jiang A Chen L Zhang X Shen X Zhou W . Machine learning algorithms for predicting the risk of fracture in patients with diabetes in China. Heliyon. (2023) 9:e18186. doi: 10.1016/j.heliyon.2023.e18186, PMID: 37501989 PMC10368844

[27] Chen Y Yang T Gao X Xu A . Hybrid deep learning model for risk prediction of fracture in patients with diabetes and osteoporosis. Front Med. (2022) 16:496–506. doi: 10.1007/s11684-021-0828-7, PMID: 34448125

[28] Shi Y Fang J Li J Yu K Zhu J Lu Y . Fracture risk prediction in diabetes patients based on Lasso feature selection and Machine Learning. Comput Methods Biomech BioMed Engin. (2026) 29(3), 511–527. doi: 10.1080/10255842.2024.2400325, PMID: 39257307

[29] Yosibash Z Trabelsi N Buchnik I Myers KW Salai M Eshed I . Hip fracture risk assessment in elderly and diabetic patients: combining autonomous finite element analysis and machine learning. J Bone Miner Res. (2023) 38(6), 876–886. doi: 10.1002/jbmr.4805, PMID: 36970838

[30] Lambin P Leijenaar RTH Deist TM Peerlings J de Jong EEC van Timmeren J . Radiomics: the bridge between medical imaging and personalized medicine. Nat Rev Clin Oncol. (2017) 14:749–62. doi: 10.1038/nrclinonc.2017.141, PMID: 28975929

[31] Lambin P Rios-Velazquez E Leijenaar R Carvalho S van Stiphout RG Granton P . Radiomics: extracting more information from medical images using advanced feature analysis. Eur J Cancer. (2012) 48:441–6. doi: 10.1016/j.ejca.2011.11.036, PMID: 22257792 PMC4533986

[32] Liu C Wei H Gong NJ Cronin M Dibb R Decker K . Quantitative susceptibility mapping: Contrast mechanisms and clinical applications. Tomography. (2015) 1(1), 3–17. doi: 10.18383/j.tom.2015.00136, PMID: 26844301 PMC4734903

[33] Guo Z Zhao M Liu Z Zheng J Gong Y Huang L . Feasibility of ultrasound radiomics based models for classification of liver fibrosis due to Schistosoma japonicum infection. PLoS Negl Trop Dis. (2024) 18(6), e0012235. doi: 10.1371/journal.pntd.0012235, PMID: 38870200 PMC11207143

[34] Ahlqvist E Prasad RB Groop L . Subtypes of type 2 diabetes determined from clinical parameters. Diabetes. (2020) 69:2086–93. doi: 10.2337/dbi20-0001, PMID: 32843567

[35] Sorokin EP Cule M Thanaj M Basty N Whitcher B Sattar N . Genetic subtypes of type 2 diabetes are distinguished through the lens of abdominal MRI. Front Genet. (2025) 16, 1605721. doi: 10.3389/fgene.2025.1605721, PMID: 40741412 PMC12307213

[36] Khan S Klochko CL Cooper S Franz B Wolf L Alessio A . Skeletal muscle ultrasound radiomics and machine learning for the earlier detection of type 2 diabetes mellitus. J Med Ultrasound. (2024) 33(2), 116–124. doi: 10.4103/jmu.jmu_12_24, PMID: 40521327 PMC12161692

[37] Ahlqvist E Storm P Käräjämäki A Martinell M Dorkhan M Carlsson A . Novel subgroups of adult-onset diabetes and their association with outcomes: a data-driven cluster analysis of six variables. Lancet Diabetes Endocrinol. (2018) 6(5), 361–369. doi: 10.1016/S2213-8587(18)30051-2, PMID: 29503172

